# Implementing and sustaining 6-month post-stroke reviews: a complexity-informed, context-sensitive programme theory for clinical practice

**DOI:** 10.3389/fstro.2026.1780242

**Published:** 2026-03-19

**Authors:** Rich Holmes, Suzanne Ackerley, Dawn Goodwin, Louise A. Connell

**Affiliations:** 1 St Richard's Hospital, University Hospitals Sussex NHS Foundation Trust, Chichester, United Kingdom; 2 Faculty of Health and Medicine, Lancaster University, Lancaster, United Kingdom; 3 Rakehead Rehabilitation Centre, East Lancashire Hospitals NHS Trust, Burnley, United Kingdom

**Keywords:** complexity analysis, follow-up care, implementation science, life after stroke, programme theory, 6-month post-stroke review, stroke rehabilitation, sustainability

## Abstract

**Introduction:**

Stroke is a leading cause of long-term disability. Six-month post-stroke reviews are recommended in multiple guidelines to identify the ongoing needs of stroke survivors and facilitate follow-up care. However, guidance on how to deliver these reviews optimally is limited. This study developed a complexity-informed, context-sensitive programme theory for the 6-month post-stroke review, clarifying its core components and producing actionable recommendations to guide implementation and sustainability across diverse contexts.

**Methods:**

The programme theory was developed from empirically derived patterns identified in a multiple case study in England, based on data collected from interest-holder interviews, observations, and documentary analysis. Context-mechanism-outcome configurations were developed heuristically through a pragmatic approach. These informed the structure and content of the programme theory and logic model. A complexity theory lens facilitated identification of multi-level system dynamics, supporting applicability across diverse contexts. The model was iteratively refined by the research team and adjusted following validation feedback from international stroke rehabilitation experts and 6-month post-stroke review interest-holders.

**Results:**

Fourteen context-mechanism-outcome configurations informed 13 core components nested within four core domains of the 6-month review: *Access & Inclusion; Identifying & Addressing Needs; Maintaining Quality;* and *System Integration*. The real-world logic model illustrates how patient-, provider-, and service-level outcomes emerge from interactions between the 6-month review and the context in which it is delivered. The resulting actionable recommendations provide guidance for implementing and sustaining 6-month post-stroke reviews in clinical practice, including flexible delivery formats, person-centered tailoring, integration across services, and strategies to enhance quality and equity.

**Conclusions:**

This study presents the first complexity-informed, context-sensitive programme theory for the 6-month post-stroke review. By translating empirically driven theory into actionable recommendations, it supports clinicians and service planners in delivering person-centered, contextually adaptable follow-up care. It also provides a foundation for future evaluation of post-stroke services internationally, enabling systematic testing of its hypothesized outcomes and adaptation across diverse healthcare settings.

## 1 Introduction

Stroke is a leading cause of death and disability worldwide (Feigin et al., 2025). At six months post-stroke, the majority of stroke survivors have new or ongoing health and social needs that require support or resolution (Lin et al., 2021). Six-month post-stroke reviews (6MRs) are recommended in UK (Intercollegiate Stroke Working Party, 2023) and European guidelines (Norrving et al., 2018) to provide a holistic assessment of stroke survivors' needs and facilitate referrals for ongoing care. However, current guidance is limited regarding how the 6MR should be delivered, how services should be structured, or how the 6MR should be integrated within the wider system. While the present study is based on services in England, the resulting programme theory provides conceptual insights for internationally comparable healthcare systems, subject to local adaptation.

Empirical studies indicate that while systematic evaluations of stroke survivors' needs can identify unmet needs, they have not been shown to consistently improve clinical outcomes (Forster et al., 2009; Ellis et al., 2010). One potential explanation for this limited evidence is the absence of a robust theoretical model describing how the 6MR produces intended clinical outcomes and which outcomes are most relevant. Without such a model, evaluations risk using measures that are not clearly linked to intended benefits, making it difficult to assess the true value of the 6MR.

Programme theory articulates how an intervention is expected to produce its intended outcomes (Weiss, 1995). Without it, the critical elements of an intervention remain unclear, leading to implementation replication that may not suit every context. Conversely, a robust programme theory clarifies the underlying mechanisms, enabling adaptation to particular contexts while still achieving intended outcomes (Funnell and Rogers, 2011). Beyond supporting implementation and sustainment, programme theory also guides selection of appropriate outcome measures for evaluating both process and effectiveness. Skivington et al. (2021) emphasize its importance for developing and evaluating complex interventions to support transferability across settings. Even after policies are implemented, it is valuable to revisit them and develop theory if one has not already been articulated (Lawless et al., 2018). This ensures that key assumptions are highlighted and uncertainties can be systematically tested (Moore et al., 2015).

Previous studies have shown wide variation in 6MR practice (Holmes R. J. et al., 2025; Abrahamson and Wilson, 2018) and incomplete implementation of policy aspirations (Abrahamson and Wilson, 2019). A contributing factor may be the absence of theory guiding the implementation, delivery, and sustainability of the 6MR. Given the heterogeneity of healthcare systems, a theoretical understanding of how context influences the delivery of the 6MR is critical for informing adaptation and evaluation within different settings.

To address this gap, this study developed a complexity-informed, context-sensitive programme theory articulating how the 6MR in England produces outcomes and how it might best be adapted in different contexts to maximize effectiveness. This study aimed to inform policy, provide a theoretical foundation for future evaluations, and generate actionable recommendations to help providers enhance the quality and effectiveness of six-month post-stroke reviews across diverse contexts.

## 2 Materials and methods

### 2.1 Project overview

The programme theory was developed through a mixed-methods project (BE MoRe: Exploring the Benefits and Expectations of the 6-Month Review for Stroke Survivors), which involved a national survey of 6MR practice within England (Holmes et al., 2025a) followed by a multiple case study exploring how outcomes are shaped by context. Findings from the multiple case study were used to develop the theoretical underpinnings presented here. The full methods of the multiple case study are described in detail elsewhere (Holmes R. J. et al., 2025), and a summary presented here for context.

The multiple case study involved three contrasting 6MR services: a nurse-led service within a community healthcare provider in the East of England; a therapist-led service within an acute healthcare provider in the South East; and a service delivered by a charitable organization in the North West. Three interest-holder groups were included across the cases: 22 service users (including stroke survivors and close family members); eight service providers (those delivering the 6MR); and six service influencers (such as regional leaders, commissioners, and managers). At each site, data were collected from interest-holder interviews, observations, and service-related documents. Data were analyzed using the Context and Implementation of Complex Interventions (CICI) framework (Pfadenhauer et al., 2017) and a complexity-informed taxonomy (Nieuwenhuijze et al., 2015). The CICI framework was used to code the data, and each contextual factor was interpreted by the research team as conceptually relevant at micro (individual), meso (service), and macro (system) levels. Interactions between contextual factors and the 6MR were mapped for each site, and patterns of interaction were developed iteratively, using the complexity taxonomy as an interpretive lens. Some factors spanned multiple levels, reflecting the complex and interdependent nature of healthcare delivery.

### 2.2 Positionality

The research was guided by a pragmatic philosophy, aiming to produce findings that are directly useful and relevant for stroke care practice (Long et al., 2018). The research team comprised two clinical academic physiotherapists with international stroke rehabilitation experience and involvement in international collaborations (LC, SA), a practicing physiotherapist with clinical leadership responsibilities (RH), and a social scientist with a background in nursing (DG). The team thus combined clinical and academic expertise in stroke rehabilitation, implementation science, and qualitative research, providing complementary perspectives that informed interpretation. Any potential bias was mitigated through monthly reflexive meetings with all team members, supplemented by additional *ad hoc* discussions as needed during theory development, ensuring interpretations remained grounded in empirical data. The programme theory was further refined through structured feedback from an international panel of academics with expertise in stroke rehabilitation and long-term support, whose perspectives provided external scrutiny of the developing model and supported consideration of its relevance beyond the UK context.

### 2.3 Generating context-mechanism-outcome configurations (CMOCs)

A brief overview of the key patterns of interaction between the 6MR and its context is provided in Table 1. These empirical findings from the original multiple case study informed the development of CMOCs. This analytic phase was not a reanalysis of the original qualitative transcripts but instead represented a higher-order synthesis of these previously identified patterns into explanatory configurations. CMOCs were used as a heuristic tool, rather than within a formal realist evaluation, and provide plausible explanatory accounts grounded in data, rather than definitive causal claims. Barriers to implementation and sustainment were derived from the research team's iterative interpretation of how contextual conditions constrained or disrupted key mechanisms. Potential strategies to address these barriers were linked to each CMOC. Strategies were selected using the sustainment-explicit Expert Recommendations for Implementing Change (ERIC) glossary (Nathan et al., 2022). Readers are directed there for definitions of the strategy terms used. All CMOCs, barriers and strategies were identified through iterative analysis of the raw data across cases and refined collaboratively within the research team.

**Table 1:** Summary of the key patterns of interaction between the 6MR and its context.

**Key patterns of interaction**	**Brief conceptual summary**
1. Access is a dynamic negotiation between service design and contextual barriers	Stroke survivors' engagement with the 6MR depends on how the structure of the service model aligns with practical, cultural, and personal circumstances. Flexible delivery methods and shared decision-making facilitated improved accessibility, whereas rigid delivery can reduce uptake and leave needs unmet.
2. Equitable service provision requires proactive adaptation	Stroke survivors have individualized needs arising from their stroke and personal circumstances. The 6MR should be tailored to meet these needs rather than standardized. Services that proactively adjust to individual needs are more likely to provide equitable care.
3. Hidden needs stay hidden unless actively unmasked	Some needs can be unidentified unless actively exposed/uncovered by the service provider. Stroke survivors at particularly risk of this include those in marginalized groups. High levels of knowledge and skills in the 6MR provider support this endeavor and the process can be optimized through the use of a flexible approach, the provision of information pre-review, and by including close family members where appropriate.
4. System levers may trigger unpredictable consequences	Policies, audits, and managerial oversight can increase uptake or efficiency but may inadvertently shift the focus away from person-centered care. Balancing process-driven metrics with a quality-driven ethos help mitigate unintended effects.
5. Outcomes are shaped by interdependence with the wider system	The 6MR's effectiveness relies on integration within the wider care system. Embedded services with shared information, coordinated pathways, and strong communication facilitate continuity, responsiveness, and tailored follow-up, whereas isolated services risk gaps and inefficiencies.

### 2.4 Developing the programme theory

The list of CMOCs was examined by the lead researcher to identify recurring patterns and conceptual overlap. Related CMOCs were grouped to form broader conceptual categories. The intended purpose of the 6MR, interpreted from interest-holder perspectives in previous work (Holmes et al., 2025b), guided the iterative development of these categories into the overarching core domains of the programme theory. The resulting core domains, along with identified barriers and implementation strategies, together shaped the structure of the programme theory.

### 2.5 Developing the logic model

A logic model was developed by the lead author, and iteratively refined by the research team, to accompany the programme theory. Drawing on complexity theory, it was designed to reflect the dynamic and context-dependent nature of the 6MR rather than assuming linear causality. The approach was informed by Mills et al.'s (2022) concept of “real-world” logic models (RWLMs). Although RWLMs offer less prescriptive guidance than linear logic models, they benefit from providing facilitators with a set of considerations to support adaptation within their unique contexts (Mills et al., 2019).

### 2.6 Validation

To validate the programme theory, multiple interest-holder consultations were undertaken during development. The key concepts of the model were checked by staff participants in the original study. A lay version was also shared with stroke survivor participants. Both the programme theory and the RWLM were checked against other 6MR services in England and reviewed by a panel of recognized international experts in stroke rehabilitation. Feedback was sought in terms of the resonance, relevance, and realism of the theory, as well as the potential transferability of the core mechanisms into other healthcare contexts. Final revisions were made in response to this feedback.

## 3 Results

### 3.1 Initial CMOCs

Overall, 14 CMOCs were developed. These can be seen in Table 2 alongside potential barriers and strategies to address each barrier. CMOCs were then interpreted to identify the underlying core components of 6MR practice. Two CMOCs related to carer/family involvement were conceptually linked and therefore combined, resulting in 13 core components. These core components were then organized into four overarching core domains: *Access and Inclusion*; *Identifying and Addressing Needs*; *Maintaining Quality*; and *System Integration*. Domains reflected the collective contribution of conceptually related core components, as interpreted by the research team. The link between CMOCs, the core components derived from them, and the domains these core components formed can be seen in Table 2.

**Table 2:** Initial CMOCs linked to core components of the programme theory.

**Core fomains**	**Core components**	**Context**	**Mechanism**	**Outcome**	**Potential barriers**	**ERIC Strategies to overcome barriers (see Nathan et al., 2022) for definitions)**	**Illustrative quote from raw data**
Access & inclusion	Enable choice through multi-format delivery methods	In the face of contextual barriers such as rurality, transport infrastructure and cost…	...providing flexible options for accessing the 6MR triggers a sense of autonomy and control, encouraging patients to engage based on personal convenience and preference…	…which is likely to increase access and uptake, and improve patient experience.	- Service adopts a one-size-fits-all model - Patient opts for ease over clinical need - Providers unaware of specific challenges relevant to local area	- Promote adaptability - Prepare patients/consumers to be active participants - Capture and share local knowledge - Conduct local needs assessment	…we're offering come to clinic, have a phone call or we'll come and see you at home […] it should all be about patient choice and the way to see the most people probably is, is offering them, you know, what works for them. *6MR Provider, Stroke Specialist Nurse, East of England*
	Adapt service provision	Certain groups (e.g., older age, greater level of disability, non-English speakers) are at risk of experiencing health inequalities…	… enabling services to adapt their provision accordingly ensures flexible and proactive care to meet the diverse needs of service users…	…which improves personalization and enhances patient experience.	- Service adopts a one-size-fits-all model - Providers unaware of individual's specific needs	- Promote adaptability - Capture and share local knowledge - Conduct local needs assessment	I think, given that each patient is so different and their needs are so different, I think there needs to be flexibility, like in appointment times and things to be able to do that because it shouldn't be about just ticking a box and saying it's been delivered. It's supposed to be meaningful and add value to the individual... *Regional leader, South East*
	Understand local population needs	Collating demographic data on patients eligible for the 6MR…	…ensures that providers are cognizant to the specific needs of their local population and are able to adapt service provision to meet these needs…	…which may enable access, increase uptake rates and reduce health inequalities.	- Providers unaware of local demographics and potential impact on access - Providers lack the ability to adapt	- Capture and share local knowledge - Conduct local needs assessment - Promote adaptability	...we've been trying to do some work with the hospitals to say the more information you can give us about the person we can make sure that our initial call is in the right language, culturally appropriate and offering the right type of support from the beginning because that's a really important element... *Regional leader. North West*
Identifying & addressing needs	Flexible timing of review	Acknowledging that stroke survivors have a unique experience following their stroke…	…negotiating the timing of the review with the stroke survivor promotes patient choice and autonomy…	…which increases the likelihood that individual needs are considered when the patient would gain the most benefit.	- Service adopts a one-size-fits-all model - Practical constraints prevent full flexibility in review timing	- Promote adaptability	I think it depends on the patient. Really does depend on the patient. Like when I, so in total I‘ve had four strokes. Two I was completely unaware of. One... so, the first one that put me in hospital, if you'd have left me for 6 months for a review after that, I'd have gone, I can't even remember half of what you're on about. *Stroke survivor, Female, North West*
	Accessible information provision	Prior to the 6MR, service users may be uncertain about the intention and process of the review…	…providing patients with clear information about its purpose helps them prepare and reflect, enabling more meaningful participation…	…which increases the likelihood that key unmet needs will be highlighted.	- Information not provided or provided in an inaccessible format for the individual - Patients do not (or are unable) to engage with information	- Intervene with patients to enhance uptake and adherence - Prepare patients to be active participants	And we send them the paperwork beforehand, so they roughly know what we're going to talk about anyway, and we ask them to do their blood pressure beforehand, and have a recent copy of their medication, so it's almost prepped. *6MR provider, South East*
	Effective skills & knowledge	When marginalized groups access the 6MR their needs may be hidden…	…equipping providers with sufficient skills and knowledge enables tailoring of care to identify and respond to diverse needs, fostering equity in service delivery…	…which increases the likelihood that specific needs are identified and addressed.	- Providers lack sufficient skills and knowledge - Service adopts a one-size-fits-all model	- Conduct ongoing training - Promote adaptability	...so, you can morph into, okay, we‘ll do BMI, we'll do temperature err blood pressure, we‘ll be scientific, we'll look and see what your results are, what your cholesterol is, so we can do that, obviously, but we can also morph into how people feel, errm what's not okay for them. *6MR provider, Stroke Specialist Nurse, East of England*
	Carer/family involvement	When the 6MR takes place in the presence of carers or family members…	…if carers are invited to participate in the review, they feel acknowledged and able to share insights and concerns. Stroke survivors feel supported, but only if their voice is prioritized and they are actively included…	…This increases the likelihood that needs are identified and that both stroke survivor and their carer feel supported.	- Carer dominates the review - Provider prioritizes carer over the stroke survivor, or uses the carer as a proxy for completing the review	- Prepare patients/consumers to be active participants - Conduct ongoing training	We had a conversation like we're having now, with all three of us in here, and I was involved as much as [*stroke survivor*] was, which was quite nice. *Husband of stroke survivor, North West*
		When the carer or family member is not present or is actively excluded from the 6MR...	…providers proactively enquiring about the carer's needs or following-up with the carer separately ensures that the carer feels recognized and supported…	…This increases the likelihood that carer needs are identified and steps are taken to rectify them.	- Carer is neglected from the review process	- Prepare patients/consumers to be active participants	There's been absolutely no support for him anywhere at all. He's just been expected to take on quite a significant change, quite a significant responsibility. All of the things I was doing, he now does, plus look after me. Plus hold down a full-time job. And for some reason his blood pressure's going up! *Stroke survivor, Female, North West*
Maintaining quality	Tailor reviews to the individual	Recognizing that the individual's own personal context (in terms of disease burden, culture, age, level of autonomy) will influence their degree of need…	…providing a personalized approach to the delivery of the 6MR ensures that it feels relevant to the patient, encouraging active engagement…	…which improves participation and experience, and enhances the quality of care received.	- Provider workload and time constraints result in superficial or “tick-box” reviews	- Obtain and use patient and family feedback	...the big thing for me is, is it doing what it's... is it meaningful for the person who's receiving the review? That, for me, that's the bottom line. *Regional leader, East of England*
	Maintain person-centered ethos	In the presence of national drivers, such as audit and financial incentives, the service may be pushed to prioritize efficiency over quality…	…maintaining a focus on person-centered outcomes buffers against metric-driven decision-making, preserving a values-based service design…	…which helps maintain quality standards and enhances patient experience.	- Staff may deprioritize patient experience in favor of key performance indicators - Reduction in patient-centered care - Variation in providers' interpretation of quality standards	- Audit and provide feedback - Develop and implement tools for quality monitoring - Obtain and use patient and family feedback - Create a learning collaborative	...so, we are kind of led by SSNAP a bit in that sense. But I don't stress over it if it doesn't fit in with what I'm doing […] as long as I feel the patient got something useful out of the session then that's, that's OK with me. *6MR provider, Stroke Specialist Nurse, East of England*
	Prioritize quality over process metrics	When there is a high degree of managerial oversight…	…a rigid focus on efficiency and cost-effectiveness may suppress innovation and reduce provider autonomy…	…which could increase uptake and rate of completion but may reduce quality and patient experience.	- Providers feel micromanaged or disempowered - Emphasis on throughput over quality	- Develop and implement tools for quality monitoring	...it's very driven to be, um, the best of everything. So, if there is a target set against it, that's slightly where the priorities are given. *6MR provider, South East*
System integration	Enable data sharing	The use of shared IT systems across services allows providers to have instant access to patient-specific information…	…which gives patients confidence that their provider understands their specific, holistic healthcare needs...	…reducing duplication and improving patient experience.	- Unable to access IT systems - Systems unable to share information across services	- Change record systems - Promote network weaving	They obviously had information from the GP and from the stroke team who'd been to see me. That seemed to be where they got the source of information, possibly also the hospital. *Stroke survivor, Male, East of England*
	Effective communication channels	Integrating the 6MR within the care pathway…	…facilitates communication between services, enhancing provider awareness of the patient's specific needs...	…which reduces repetition and improves patient experience.	- Siloed services - Limited sharing of information	- Promote network weaving - Build a coalition	...we saw it very much as an integrated pathway that we were considering the patient and the patient's journey through the acute, the rehab, ESD [*early supported discharge*], potentially, and then 6-month follow-up. So, I think it is part of the integration of the journey rather than having separate pockets of work. *Regional leader, East of England*
	Optimize referral processes & pathway flow	Where healthcare services are functioning with limited resources and there is a critical need to deliver services cost-effectively…	…if providers have the sufficient skills and knowledge to assess needs thoroughly and are able to rectify unmet needs or, when necessary, appropriately refer onward, rather than defaulting to the GP…	…then there is likely to be reduced burden on other healthcare services, more efficient use of system resources, and an improved experience for the stroke survivor as they move through the system.	- Responsibility for onward referral is shifted to a third party i.e., GP - Poor referral processes - Provider workload and time constraints result in a need to shift responsibility to other services	- Conduct ongoing training - Promote network weaving - Build a coalition	So, sometimes it's just that reassurance and confidence building that I think is invaluable and then you'd hope that they wouldn't […] be going back to their primary... you know to the GP or whoever else with anxiety or worry which is a lot of you know what we see at 6 months because they haven't had that opportunity to be reassured and actually what they're experiencing is all part of their recovery. *6MR provider, Stroke Specialist Nurse, East of England*

### 3.2 Summary of programme theory

**Figure 1:**
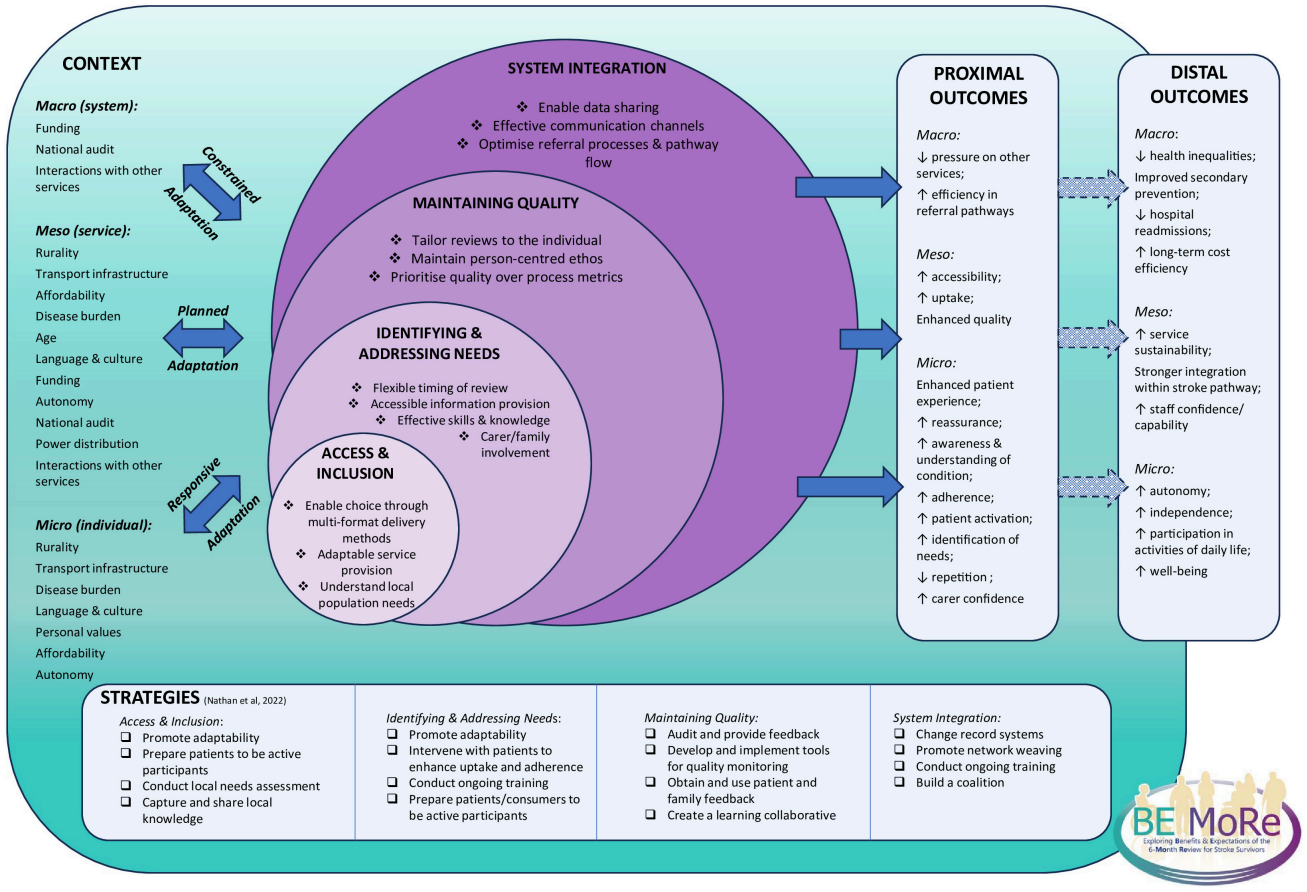
“Real-world” logic model illustrating the programme theory. The four core domains contain the core components of the 6MR. Contextual factors shape the delivery of the 6MR at different levels. To ensure fidelity to the core components, services must be able to adapt, though the type of adaptation differs at different levels of context. This ongoing interaction influences if and how the proximal outcomes are realized. Proximal outcomes may then contribute to the emergence of distal outcomes. Successful delivery in each core domain can be facilitated using implementation and sustainment strategies.

The programme theory is structured around the four core domains (containing the core components of the 6MR), the key contextual factors that interact with the 6MR, the suggested strategies to facilitate implementation and sustainment, and the hypothesized outcomes. A more detailed version of the programme theory can be found in Supplementary File 1. The associated RWLM illustrating the interacting elements can be seen in Figure 1.

Within the programme theory, the intended purpose of the 6MR is articulated as providing a person-centered, needs-based follow-up that supports individuals as they navigate life after stroke by offering advice, education and reassurance, and, where available and appropriate, connects them to relevant services or resources. This intended purpose frames the four core domains, which function in a semi-hierarchical but interdependent manner. These domains interact dynamically with contextual factors at the macro, meso, and micro level, shaping both the delivery of the 6MR and the outcomes it can achieve.

*Access and Inclusion* concerns the extent to which contextual barriers impact two complementary but distinct aspects of engagement with the 6MR. Access relates to whether individuals can engage with the review in the first instance, facilitated through flexible delivery formats and patient choice. Inclusion relates to how the review is experienced once accessed, requiring adaptation of delivery in response to language, cultural, and individual needs so that diverse populations feel acknowledged and able to participate meaningfully.

*Identifying and Addressing Needs* focuses on uncovering the full range of stroke survivor needs, some of which may remain hidden without active identification by the 6MR provider. Personalized reviews, skilled providers, appropriate pre-review information, and involvement of family or carers support more comprehensive identification and facilitate matched care.

*Maintaining Quality* relates to delivering a person-centered review that results in tangible benefit to the stroke survivor. The review should extend beyond a procedural exercise, with follow-up where necessary to ensure actions are completed and outcomes realized. Quality is strengthened through locally relevant data capture and quality improvement initiatives.

*System Integration* relates to how effectively the 6MR operates within the wider pathway. When well-integrated, the 6MR can make effective referrals, reduce duplication, and improve patient flow into services they need. Shared information systems and strong intra-system relationships were identified as key enablers; poor integration risks fragmented care and reduced service user satisfaction.

Context, interacting with the 6MR at micro, meso, and macro levels, shapes how each domain can function. Some contextual factors influenced across multiple levels. Readers are directed to our previous work for a more expansive overview of the relevant contextual factors displayed in the RWLM (Holmes R. J. et al., 2025). Importantly, the 6MR was found to be more effective when services were able to adapt delivery to contextual conditions, but the nature of this adaptation differed across levels. At the micro level, an individual's ability to access and effectively participate in the review is shaped by multiple factors related to their individual circumstances and the environment they are situated in. Here, *responsive adaptation* is required to ensure equitable participation and that needs are met. At the meso level, contextual factors influence how services are configured and delivered. Services should engage in *planned adaptation* to respond to gradual changes in the local population over time while balancing service and system constraints with person-center care. Macro-level contextual factors set the limits within which the services operate, often necessitating *constrained adaptation*, where services adjust practices to comply with external mandates while trying to mitigate unintended consequences.

The RWLM also articulates strategies aligned to each domain to support implementation and sustainment efforts. Guided by the intended purpose of the 6MR, outcomes are hypothesized at the micro, meso, and macro levels and conceptualized as proximal (directly linked to 6MR delivery) and distal (emerging over time through wider system interactions), illustrating both the immediate and potential long-term impacts of the 6MR.

### 3.3 Validation

Detailed feedback on the programme theory and RWLM was received from nine stroke rehabilitation experts, representing a range of multi-disciplinary professions, from the UK, Sweden, Germany and Canada, and was further validated by services in two additional regions of England, complementing the three regions included in the original case studies. Feedback confirmed that the overall programme theory and RWLM were credible, comprehensive, and reflected the complexity of the 6MR in practice. A consistent theme in the feedback related to the timing of the 6MR. Experts agreed with the need for flexibility to match individual need, however, they expressed concerns that removing the “six-month” label could risk the review becoming de-prioritized, given how embedded the 6MR is within policy and audit requirements. The *Discussion* below reflects this ongoing debate. Feedback also highlighted a need for clearer articulation of contextual factors and outcomes; this informed the final iteration of the programme theory and the RWLM. Additionally, experts outside of the UK highlighted that certain contextual factors, such as national audits and funding pathways, may not be directly replicable in their countries, indicating that application of the theory elsewhere may require additional testing.

## 4 Discussion

This article presents the first complexity-informed, context-sensitive programme theory for the 6MR for stroke survivors. Drawing on empirical findings and expert validation, the programme theory articulates how context interacts with the 6MR to produce outcomes. Through a logic model that acknowledges real-world complexity, it provides a conceptual bridge for researchers evaluating the 6MR and a guide for those implementing and sustaining 6MR services within complex systems in diverse and evolving contexts. Importantly, these theoretical insights have been translated into actionable recommendations for clinical practice to support real-world service delivery. In the UK context, where stroke services are under pressure to manage increasing patient volumes with finite resources, the programme theory provides a framework to enhance efficiency and effectiveness of 6MR provision. By tailoring delivery to individual need, streamlining referrals, and ensuring more relevant follow-up, services can optimize resource use while supporting person-centered care.

Until now, the 6MR has lacked a programme theory clearly articulating its core components, presented here across four domains progressing from micro to macro levels. Applying these core components in practice relies on balancing intervention fidelity with adaptability, a topic often debated in implementation research (Von Thiele Schwarz et al., 2021; Albers et al., 2024; Fixsen, 2025). Attaining this balance is especially relevant when implementing complex interventions in complex systems where rigid adherence to a model risks failure if variations in context are not acknowledged (Murdoch et al., 2023; May et al., 2016). When variations in context demand variations in intervention delivery, Hawe (2015) suggests that fidelity can be maintained by standardizing in terms of *function* rather than *form*. As such, 6MR providers should strive to deliver the *functions* described in the core component domains while acknowledging that how they are delivered can be flexible. Doing so enables the principles of 6MR implementation to remain consistent, while recognizing that local contextual factors will determine the most appropriate form of delivery. To support clarity for service providers, these core components have been translated into key actionable recommendations (see Box 1). Further detail regarding each recommendation is available in Supplementary File 2.

Box 1 **Actionable recommendations for 6-month review provision.**

1. **Offer stroke survivors a choice** of how and where they might access the review (e.g., virtual, in-person, home-based).
2. **Tailor the timing of the review** to the stroke survivor's readiness and recovery stage.
3. **Provide clear, accessible information** in advance so that the stroke survivor can prepare for the review.
4. **Collect and review demographic data** to identify groups with reduced access to the review and develop targeted approaches to increase uptake.
5. **Involve carers and family members meaningfully**, while keeping the stroke survivor's voice central.
6. **Tailor the review's content and delivery** to individual need (considering age, level of disability, language, culture etc.).
7. **Ensure staff has ongoing training, development and support** to be able to effectively identify and respond to diverse needs.
8. **Prioritize the stroke survivor's goals, wishes and expectations** over meeting process metrics or checklist completion.
9. **Enable information sharing across services** using integrated information systems, shared records, or agreed communication channels.
10. **Establish strong links with system partners** to facilitate direct referrals rather than defaulting to primary care services.

A key aspect of these recommendations is the inclusion of personalization as a central tenet. This aligns with other research on 6MRs which emphasized the importance of flexibility within the timing and format of the review process (Abrahamson and Wilson, 2019; Freeman et al., 2024). Implementing a tailored approach to the timing of reviews is inherently challenging. Ensuring a shared decision-making approach and greater system integration to facilitate information sharing may help to achieve this ambition. Furthermore, countering a one-size-fits-all approach, it should be considered that a single review may be insufficient to fully appreciate the needs of all stroke survivors, and that follow-up may be warranted in some situations. Such recommendations must, however, be balanced alongside the financial and capacity restraints of the system. Implementing this level of individualization would likely require service and system reorganization and a shift in current mindsets, including reconsideration of how post-stroke reviews are conceptualized and named in the future.

The developed programme theory emphasizes the need to adapt the delivery of the 6MR, in response to context, so that its function is optimized. Adaptation, however, is applied differently across each level of the system. This multi-level framing aligns with a growing body of evidence viewing adaptation as an inherent and necessary feature of implementing and sustaining complex interventions (Chambers et al., 2013; Lyng et al., 2021; Moore et al., 2021). At the individual level, providers require sufficient flexibility within their service to adapt to meet the specific needs of each stroke survivor (*responsive adaptation*). Doing so ensures a personalized experience and increases the likelihood that needs are highlighted and addressed. Adaptation at this level does not involve changes to service structure; rather, it is built into the delivery model. In contrast, at the meso level, adapting a service by reactively responding to a variable context could create chaos and limit its sustainability. Instead, services require *planned adaptation* through a proactive and strategic approach. This may be achieved through effective data collection and a heightened awareness of changes within the local context, allowing the service to evolve over time. At the macro level, this longer-term adaptation is subject to the limitations of national level drivers and it may be necessary to consider the needs of the local population alongside these constraints (*constrained adaptation*) acknowledging that resources are not infinite.

This layered view of requisite adaptation offers a novel conceptualization supporting a targeted approach based on the aspect of the system under consideration. In essence, the programme theory highlights the dynamic interaction between context and intervention, suggesting that as context continually evolves, so too must service delivery. By increasing the understanding of how services within complex systems might attempt to adapt across contexts, providers may be guided to ensure the resilience and sustainability of their 6MR service provision (Lyng et al., 2021).

### 4.1 Strengths and limitations

The programme theory is strengthened by being developed and refined through rigorous qualitative research on three contrasting organizational models (Holmes R. J. et al., 2025) and validated by experts in stroke rehabilitation. The associated RWLM is complexity-informed reflecting the non-linear and emergent nature of outcomes. This approach provides a framework for adapting services in response to contextual factors, supporting the implementation and sustainability of the 6MR. However, the programme theory is limited in that outcomes remain hypothesized based on the assumptions of interest-holders rather than through empirical testing, reflecting constraints in longitudinal follow-up in the original study. Implementers of the model should treat the stated outcomes as proposed expectations rather than a guarantee of effect. Local evaluation and adaptation are recommended to ensure that intended mechanisms produce desired outcomes in each service context.

The multiple case study approach ensured an in-depth exploration of context at multiple levels. However, the use of a higher level of abstraction inevitably distances the resulting conceptual claims from the empirical data, potentially obscuring contextual nuances. It is anticipated that concepts generated in this programme theory may be transferable to other healthcare settings. However, applying this theory to healthcare models outside the UK may require consideration of additional contextual factors, such as wider cultural influences and healthcare funding routes. While a positive level of engagement was received from rehabilitation experts, it was not feasible to gather feedback from the full spectrum of international healthcare systems. The programme theory is therefore presented as a flexible and evolving framework, intended to guide implementation in other contexts while being adapted over time to reflect local conditions and emerging evidence.

Finally, despite using multiple data collection methods and engaging a range of interest-holders, some groups may remain under-represented. Consequently, important areas of context that impact under-represented groups may be missing in the current theoretical understanding.

## 5 Conclusion

This article presents a novel, complexity-informed and context-sensitive programme theory articulating the core components of the 6MR. By emphasizing how context interacts with the 6MR across multiple levels, it offers a framework for understanding how outcomes are produced in practice. The theory highlights adaptation as an essential, multi-level process, contributing to broader discussions on delivering and sustaining complex interventions within complex systems.

The theory provides researchers with a foundation for designing and implementing evaluations of the 6MR in England and for guiding implementation of post-stroke follow-up care practices in other countries. Future work should empirically test the hypothesized outcomes and examine wider system impacts, including economic evaluations. By translating this theory into actionable recommendations, it serves as a practical guide for 6MR providers to design, deliver, refine and sustain the 6MR across diverse settings. In doing so, the theory supports more resilient, contextually attuned, and person-centered approaches to post-stroke review and recovery.

## Data Availability

Publicly available datasets were analyzed in this study. This data can be found here: doi: https://doi.org/10.21203/rs.3.rs-7490223/v1.

## Supplementary material

Supplementary File 1 Extended version of programme theory.

Supplementary File 2 Extended version of actionable recommendations.
