# Supplementary material for: Implementing and sustaining 6-month post-stroke reviews: a complexity-informed, context-sensitive programme theory for clinical practice
Source: Front Stroke. 2026 Mar 19;5:1780242. doi: 10.3389/fstro.2026.1780242 (PMC13045055; doi:10.3389/fstro.2026.1780242)
Supplement: Supplementary File 1 — Extended version of programme theory. [file Supplementary_file_1.docx]

**Supplementary 1. Programme Theory**

**Programme Theory for the Six-Month Post-Stroke Review (6MR)**

The following programme theory articulates the core components that should be included in the 6MR, describes the key contextual factors that interact with it, and outlines the hypothesised outcomes. Within this programme theory, the intended purpose of the 6MR is articulated as providing a person-centred, needs-based follow-up that supports individuals as they navigate life after stroke by offering advice, education and reassurance, and, where available and appropriate, connects them to relevant services or resources. In addition, the 6MR contributes to system-level learning by generating information on service-use and post-stroke needs, which can inform future service planning and quality improvement. This purpose provides the conceptual anchor for the development of the four core domains and hypothesised outcomes. The programme theory should be read in conjunction with the ‘real-world’ logic model (RWLM) included in the main article.

**Core Components**

The core components of the 6MR are described across four nested domains, illustrated in the RWLM in a semi-hierarchical fashion. The term ‘semi-hierarchical’ reflects that while the domains are organised progressively, from individual-level through to system-level, each retains some capacity to operate independently. Outer domains can still operate if inner domains are underperforming, though often with reduced effectiveness. For example, a service may excel at *Maintaining Quality* but only for a limited cohort of stroke survivors if *Access and Inclusion* is weak.

*Access and Inclusion:* Findings indicate that access to the 6MR can be limited by contextual barriers. Policy mandates that all stroke survivors should receive a 6MR. However, they do not need to receive it in the same way. A one-size-fits-all approach inhibits access for some individuals. Services should offer multiple formats, including face-to-face, telephone or virtual, to enable patient accessibility. However, this should be negotiated with the stroke survivor to ensure suitability.

Services should be agile in responding to the specific needs of individuals. Considerations include making provisions for non-English speakers, ensuring stroke survivors in care homes are able to participate, and ensuring costs to access the service are minimised to prevent health inequities. The function in this domain should be to optimise accessibility and ensure inclusivity. Understanding the specific needs of the local population through data collection supports this ambition.

*Identifying and Addressing Needs:* This domain focuses on ensuring that all relevant needs are identified and effectively addressed. Data indicate that needs often remain hidden and require an active process to uncover. To achieve this, a core component is the effective provision of information in accessible formats prior to the review, allowing service users the opportunity to prepare and reflect on what is important to them.

Equally important is that the 6MR is personalised to the individual to ensure the full range of issues are captured. Potential needs to be addressed include, but are not limited to, physical impairments and functional limitations; cognitive, emotional, and psychosocial difficulties; communication problems; fatigue and sleep disturbance; pain and spasticity; financial and benefits advice; vocational and return-to-work support; support for family and carer roles; lifestyle and secondary prevention needs; medication review; driving and transport advice; sexual function and intimacy concerns; and signposting to social reintegration, community participation, and peer support.

The identification of needs relies heavily on the provider’s skills and knowledge to be able to adapt the review to each individual and adeptly uncover hidden needs. Family members, particularly informal carers, should be involved where possible, to support the unmasking of needs and ensure a comprehensive understanding of the stroke survivor’s situation.

*Maintaining Quality:* Process-driven measures of efficiency should not be the service’s primary target. Services should maintain a person-centred ethos, placing the stroke survivor at the heart of all they do. Alongside this, a core component of the 6MR is ensuring the review translates into tangible improvements in care and quality of life. This may necessitate a need to follow-up to ensure outcomes have been achieved as intended.

*System Integration:* The 6MR’s impact is maximised when it connects efficiently to other parts of the pathway, supporting optimal use of healthcare resources. A key responsibility of the 6MR is to act as a gatekeeper to other services, ensuring referrals are made responsibly, considering cost-effectiveness and the need to avoid exacerbating pressures elsewhere in the system.

The 6MR achieves its greatest value when fully integrated within the broader system. This includes making best use of shared IT systems where possible, enabling integrated data sharing, and ensuring regular opportunities for communication across services. Without this integration, there is a greater risk of unidentified and unmet needs, duplication of work, and fragmented care. By supporting efficient referrals and optimised patient flow, the 6MR can strengthen the pathway as a whole.

**Context**

Context is not merely the backdrop of the 6MR but an overarching, entwined, and integral part of how it functions. In the RWLM, context is represented as all-encompassing rather than as a separate entity, with bidirectional arrows illustrating the dynamic interactions between context and the intervention. Data showed that these key interactions happen at different levels: *Micro* (related to the delivery of the 6MR to the individual), *Meso* (related to the service structure and operation), and *Macro* (related to the wider system). Analysis showed that the 6MR functions best when able to adapt to context, but the nature of requisite adaptation differs across levels: at the micro level, services must engage in *responsive adaptation* to meet the needs of individuals; at the meso level, *planned adaptation* is required to organise services around local conditions and resources; and at the macro level, services are often subject to *constrained adaptation*, responding to national drivers and system-level constraints.

*Micro:* A stroke survivor’s engagement is shaped by contextual factors such as rurality, transport infrastructure, affordability, personal values, language, and cultural diversity. These influence an individual’s ability to access and participate in the review, as well as their preferred delivery mode.

Certain groups are at higher risk of experiencing inequitable engagement. Stroke survivors who have greater disease burden, are care home residents, or those with language and cultural needs that are not accommodated may find it more difficult to benefit fully from the 6MR. Inequities are most pronounced where services cannot adapt provision to individual circumstances.

*Meso:* The organisation and delivery of the 6MR reflects an interplay between contextual conditions and organisational levers. Rurality, transport infrastructure, and affordability shape decisions about service location, format, and outreach capability, while local demographic needs inform organisational planning, resource allocation and service structure. Funding arrangements, audit requirements, and performance targets act as levers that guide priorities, sometimes generating tension between efficiency and person-centred care. Power dynamics between providers and decision-makers may also complicate the service’s ability to adapt and innovate. Additionally, relationships with other services in the pathway can determine how follow-up responsibilities are shared and what provisions are available to stroke survivors, further influencing 6MR structure and content.

*Macro:* National-level drivers, including funding arrangements and audit requirements, set the parameters within which services operate. These levers can raise the profile of the 6MR and encourage uptake, but may also generate unintended consequences, such as incentivising standardisation over personalisation. Affordability at the system level remains a key determinant of the scope and sustainability of provision, with interactions between national drivers and service-level practices either facilitating or constraining equitable access for stroke survivors.

**Strategies**

Strategies to support 6MR implementation and sustainment are presented within the RWLM, organised by the four core domains to help services target specific areas of challenge. The suggested strategies serve a dual purpose: to leverage contextual factors that facilitate 6MR delivery and to mitigate factors that act as barriers. While strategies were informed by empirical findings, services may require tailored approaches to meet local needs effectively.

*Access and inclusion:* This domain can be supported by collecting information on the needs of the local population, helping services adapt delivery to support those needs and optimise inclusion. Adaptability is crucial to enable responsiveness to the wide range of post-stroke presentations encountered. Access can be further enhanced through proactive strategies to engage the local stroke community, promote the purpose of the 6MR and enable shared decision-making.

*Identifying and Addressing Needs:* Unmasking needs requires an individualised, rather than a standardised, approach. To achieve this, providers benefit from ongoing training and development to strengthen skills in identifying issues and recognising patterns. Efficiency can be improved by encouraging stroke survivors to actively identify their own health and social care needs. The level of support required to facilitate this process will vary between individuals, highlighting the need for a flexible approach to delivery.

*Maintaining Quality:* Strategies to ensure success in this domain are centred on effective data capture to guide service delivery. Information regarding service delivery should be regularly collected and responded to through quality improvement initiatives. To support this, local tools should be developed to systematically capture and monitor quality metrics. Providers should also actively collect qualitative data from service users to ensure the service remains relevant and acceptable to them.

*System Integration:* Achieving system integration requires building and maintaining strong relationships with other services within the system, promoting coordinated responsibilities and continuity of care. By fostering these connections, providers may identify opportunities to streamline pathways, reduce duplication and improve cost efficiency. Effective integration can be supported through shared information systems. Providers should consider whether their current or intended information systems sufficiently enable the effective information transfer between services.

**Outcomes**

Outcomes associated with the 6MR are described as proximal and distal. Proximal outcomes are closely linked with, and directly resulting from, the 6MR, and are shaped by context across micro, meso and macro levels.

Distal outcomes emerge over time through wider system interactions and are less directly attributable. In the RWLM, these are depicted as extending beyond the ‘boundary’ of 6MR-related context. Broken lines connecting proximal to distal outcomes indicate a non-linear relationship that lacks direct causal effect. Distinguishing between proximal and distal outcomes informs the selection of realistic outcomes for future evaluations, and avoids over-claiming what the 6MR can achieve in isolation.

*Micro:* Proximal outcomes at the micro level relate to the direct impact to individuals accessing the 6MR. Effective delivery enhances service user experience, reduces unnecessary repetition, fosters a sense of reassurance, and strengthens confidence in ongoing management. Proactive identification of unmet needs reduces inequitable experiences and heightens the likelihood that those needs are met through matched care and appropriate referrals. Accessible information provision and active engagement promote stroke survivors’ understanding of their condition and of secondary prevention strategies. These proximal gains lay the foundations for more distal outcomes such as increased autonomy, independence and participation in daily activities.

*Meso:* Proximal outcomes at the meso level focus on service performance including improved accessibility, uptake and quality. These are dependent on the service’s ability to adapt in response to local context. Over time, these outcomes may enable sustainable service provision and stronger pathway integration.

*Macro:* Outcomes at this level relate to system performance and equity. Proximally, the 6MR can reduce pressure on other services by addressing needs earlier and streamlining referrals. Over time, as services consistently adapt service provision to diverse needs and further integrate into pathways, they contribute to distal outcomes such as reducing health inequalities. This relies on sustained provision, where cumulative effects of improved access, quality, and integration enable more equitable distribution of post-stroke support. Ultimately, these mechanisms may underpin wider system efficiencies, improve secondary prevention, reduce hospital readmissions, and improve recovery trajectories for stroke survivors.
