# Supplementary material for: Implementing and sustaining 6-month post-stroke reviews: a complexity-informed, context-sensitive programme theory for clinical practice
Source: Front Stroke. 2026 Mar 19;5:1780242. doi: 10.3389/fstro.2026.1780242 (PMC13045055; doi:10.3389/fstro.2026.1780242)
Supplement: Supplementary File 2 — Extended version of actionable recommendations. [file Supplementary_file_2.docx]

**Supplementary 2. Actionable Recommendations**

| **Actionable Recommendations for Six-Month Review Provision** |
| --- |
| 1. **Offer stroke survivors a choice** |
| Stroke survivors should be offered a choice of how and where they access the 6MR. This should involve shared decision making between the stroke survivor and the provider to ensure the method matches the needs of the individual. Where possible, stroke survivors should be seen face-to-face (either in-person or virtually) to optimise the unmasking of needs. However, on occasions where the provider is clear there is minimal need or if the stroke survivor has a clear preference, then telephone reviews may be appropriate. |
| 1. **Tailor the timing of the review** |
| While six months suits the majority of stroke survivors, it is not the optimal time for all. The timing of the review should be a shared decision between the provider and the stroke survivor and based on when they are likely to get the optimal benefit. This is an individualised decision based on the stroke survivor’s personal context and does not seem to follow a predictable pattern. When the stroke survivor consents, the views of carers and family members should be considered in this decision. |
| 1. **Provide clear, accessible information** |
| The purpose should be clearly articulated to the stroke survivor in advance of the 6MR. Stroke survivors should have enough information to enable them to prepare for the review so that they are able to consider needs they wish to highlight. Providers should ensure that information given to stroke survivors is in an accessible format, considering those that may have communication difficulties or may speak a language other than English. |
| 1. **Collect and review demographic data** |
| The 6MR should be offered to all stroke survivors. Providers should routinely collect and analyse demographic data of those accessing, and those not accessing, their service. They should look for patterns in those not accessing the review to understand barriers to access and to attempt targeted approaches where appropriate. |
| 1. **Involve carers and family members meaningfully** |
| Informal carers and close family members may have insights that enable certain issues to be raised in addition to those highlighted by the stroke survivor. The involvement of carers and family members in the review process is appreciated and valued. However, care should be taken to ensure the stroke survivor remains central to the process. Using family members as proxies is not encouraged unless absolutely necessary and all attempts should be made to include the stroke survivor (including when the stroke survivor resides in a care home). |
| 1. **Tailor the review’s content and delivery** |
| Stroke survivors have unique needs and unique contexts that shape their experience. They require a personalised review to ensure their needs are unmasked. Tailoring the 6MR in terms of content and how it is delivered is important to ensure the stroke survivor achieves optimal outcomes. Evidenced based tools can support this process by providing a useful structure to the review, particularly for less experienced reviewers. However, they should serve as guides rather than rigid templates. Tools should be used flexibly to support, rather than inhibit, personalised care. |
| 1. **Ensure staff have ongoing training, development and support** |
| Those who deliver the 6MR should have access to ongoing development opportunities. Providing the 6MR often involves lone-working in emotionally-challenging situations. Providers benefit from regular peer support for pastoral care as well as opportunities to discuss cases to support their learning. |
| 1. **Prioritise the stroke survivor’s goals, wishes and expectations** |
| In the presence of external pressures, providers should strive to keep the stroke survivor at the heart of the review process, maintaining a person-centred ethos to service delivery. This should be prioritised over process-driven metrics while acknowledging that these metrics are important at a system-level. The 6MR should not be adapted purely to meet targets on these metrics at the expense of person-centred care. |
| 1. **Enable information sharing across services** |
| Some 6MR services will find this very easy if they are already integrated within a stroke service. For those that provide 6MRs as a stand-alone service, they will need to ensure effective methods are in place that enable the transfer of information in an efficient and protected way. Doing so will optimise referral pathways in to the 6MR and ensure providers are aware of the individual stroke survivor’s journey, preventing patients from having to repeat information. Using shared IT systems across the pathway will be the most effective way to achieve this. |
| 1. **Establish strong links with system partners** |
| Building connections with system partners expands the providers’ awareness of available services they can refer stroke survivors into and optimises the referral pathways. Providers should avoid adding the responsibility of onward referrals to other healthcare providers, particularly those in primary care. |
